# Identification of a High-Frequency Intrahost SARS-CoV-2 Spike Variant with Enhanced Cytopathic and Fusogenic Effects

**DOI:** 10.1128/mBio.00788-21

**Published:** 2021-06-29

**Authors:** Lynda Rocheleau, Geneviève Laroche, Kathy Fu, Corina M. Stewart, Abdulhamid O. Mohamud, Marceline Côté, Patrick M. Giguère, Marc-André Langlois, Martin Pelchat

**Affiliations:** a Department of Biochemistry, Microbiology and Immunology, Faculty of Medicine, University of Ottawa, Ottawa, Ontario, Canada; b uOttawa Center for Infection, Immunity and Inflammation (CI3), Ottawa, Ontario, Canada; c University of Ottawa Brain and Mind Research Institute, University of Ottawa, Ottawa, Ontario, Canada; d Ottawa Institute of Systems Biology, University of Ottawa, Ottawa, Ontario, Canada; Medical School, National and Kapodistrian University of Athens

**Keywords:** COVID-19, SARS-CoV-2, syncytia, genetic variants, high-throughput sequencing, spike protein

## Abstract

The severe acute respiratory syndrome coronavirus 2 (SARS-CoV-2) is a virus that is continuously evolving. Although its RNA-dependent RNA polymerase exhibits some exonuclease proofreading activity, viral sequence diversity can be produced by replication errors and host factors. A diversity of genetic variants can be observed in the intrahost viral population structure of infected individuals. Most mutations will follow a neutral molecular evolution and will not make significant contributions to variations within and between infected hosts. Herein, we profiled the intrasample genetic diversity of SARS-CoV-2 variants, also known as quasispecies, using high-throughput sequencing data sets from 15,289 infected individuals and infected cell lines. Despite high mutational background, we identified recurrent intragenetic variable positions in the samples analyzed, including several positions at the end of the gene encoding the viral spike (S) protein. Strikingly, we observed a high frequency of C→A missense mutations resulting in the S protein lacking the last 20 amino acids (SΔ20). We found that this truncated S protein undergoes increased processing and increased syncytium formation, presumably due to escaping M protein retention in intracellular compartments. Our findings suggest the emergence of a high-frequency viral sublineage that is not horizontally transmitted but potentially involved in intrahost disease cytopathic effects.

## INTRODUCTION

Observed for the first time in 2019, the severe acute respiratory syndrome coronavirus 2 (SARS-CoV-2) and its associated disease, COVID-19, have caused significant worldwide mortality and unprecedented economic burdens. SARS-CoV-2 is an enveloped virus with a nonsegmented, positive-sense, single-stranded viral RNA (vRNA) genome comprised of ∼30,000 nucleotides (1, 2). The virus is composed of four main structural proteins, encoded in the last 3′-terminal third of the viral genome: the spike glycoprotein (S), membrane (M), envelope (E), and nucleocapsid (N) (3–5). Attachment to the host receptor angiotensin-converting enzyme 2 (ACE2) is mediated by the S protein expressed on the surface of the virion (6). Following its association, the S protein is cleaved into two separate polypeptides (S1 and S2), which triggers the fusion of the viral particle with the cellular membrane (6, 7). Once inside a cell, its RNA-dependent RNA polymerase (RdRp), which is encoded in the first open reading frame of the viral genome (8), carries out transcription and replication of the vRNA genome. In addition, mRNAs coding for the structural proteins (e.g., S, M, E, and N) are expressed by subgenomic RNAs (8). Once translated, the S, M, and E proteins localize and accumulate at the CoV budding site in the endoplasmic reticulum (ER)-Golgi intermediate compartment (ERGIC) (9). One aspect of CoV biology is that CoV virions bud into the lumen of the secretory pathway at the ERGIC and must then traffic through the Golgi complex and anterograde system to be efficiently released from host cells (10). The S protein possesses an endoplasmic reticulum retrieval signal (ERRS) at its carboxy terminus, which is required for trafficking through the ERGIC (11). At this location, the spike protein interacts with the M protein, which has been shown to be essential for accumulation at the ERGIC. The N protein then associates with the viral genome and assembles into virions, which are transported along the endosomal network and released by exocytosis (8). If not retained at ERGIC, the S protein traffics through the Golgi complex and is preactivated by resident proteases prior to reaching the plasma membrane. Here, it can mediate cell fusion between adjacent cells, resulting in the production of multinucleated cells, or syncytia (7, 12, 13).

Genomic sequencing of SARS-CoV-2 vRNA from infected populations has demonstrated genetic heterogeneity (14–20). Several recurrent mutations have been identified in consensus sequences, and the geographical distribution of clades has been established. Because they induce an abundance of missense rather than synonymous or nonsense mutations, it was suggested that regions of the SARS-CoV-2 genome were actively evolving and might contribute to pandemic spreading (20). It was observed that variations are mainly comprised of transition mutations (purine→purine or pyrimidine→pyrimidine), with a prevalence of C→U transitions, and might occur within a sequence context reminiscent of APOBEC-mediated deamination (i.e., [AU]C[AU]) (21, 22). Consequently, it was proposed that host editing enzymes might be involved in coronavirus genome editing (23, 24).

Transmitted genomes and consensus sequences are only part of the genetic landscape with regard to RNA viruses. Replication of RNA viruses typically produces quasispecies in which the transmitted viral RNA genomes do not exist as a single sequence entity but rather as a population of genetic variants (25). These mutations are most frequently caused by both the error-prone nature of each of their respective viral RdRps and the host RNA editing enzymes, such as APOBECs and ADARs (26). However, the RdRp complex of large RNA viruses, such as coronaviruses, sometimes possesses exonuclease proofreading activity, and consequently, they have lower error rates (25, 27). Quasispecies may sometimes exhibit diminished replicative fitness or deleterious mutations and exert different roles that are not directly linked to viral genomic propagation (28). Mutations that form the intrahost genetic spectrum have been shown to help viruses evade cytotoxic T cell recognition and neutralizing antibodies, rendering these viruses more resistant to antiviral drugs (28). Additionally, these mutations can also be involved in modulating the virulence and transmissibility of the quasispecies (28).

In this study, we focused on assessing intrahost genetic variations of SARS-CoV-2. We analyzed high-throughput sequencing data sets to profile the sequence diversity of SARS-CoV-2 variants within distinct sample populations. We observed high intrahost genetic variability of the viral genome. By comparing variation profiles between samples from different donors and cell lines, we identified highly conserved subspecies that independently and recurrently arose in different data sets and, therefore, in different individuals. We further analyzed the dominant variant SΔ20 in a functional assay and demonstrate that this truncated S protein avoids inhibition caused by M protein and enhances syncytium formation. We provide evidence for the existence of a consistently emerging variant identified across geographical regions that may influence intrahost SARS-CoV-2 pathogenicity.

## RESULTS

### High intragenetic variability of the SARS-CoV-2 genome in infected individuals.

**FIG 1:**
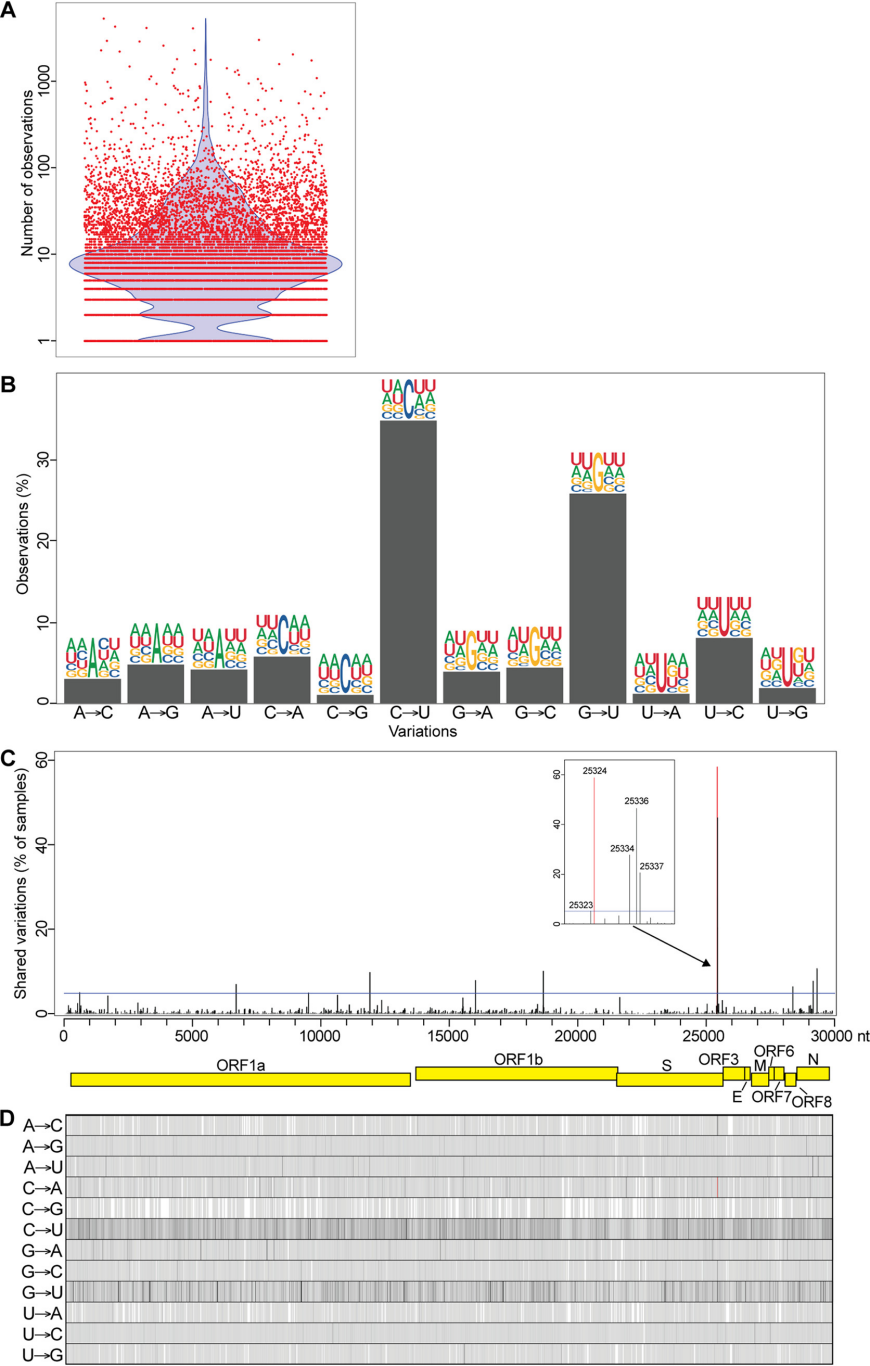
Intrasample variability of the SARS-CoV-2 genome in infected individuals. (A) Number of intragenetic variations observed for each sample analyzed. The red dots represent the 11,362 samples analyzed, and the blue violon shows the distribution of the data. (B) Type of variation and sequence context for each intrasample variable position. Bars represent the percentage of each type. Sequence context is represented by logos comprised of the consensus nucleotides (center) with 2 nt upstream and 2 downstream from each intrasample variable position. (C) Recurrent intragenetic variations are represented as percentages of samples containing variations at each position. The SARS-CoV-2 genome and its genes are represented by yellow boxes below the graph. The blue line indicates 5% shared variations and was used to extract the recurrent intrasample variations listed in Table 1. The inset represents a magnification of the cluster identified at the end of the S gene. (D) One-dimensional representation of the data shown in panel C for each type of variation individually. The location of the C→A variation at position 25,324 is indicated by a red line in panels C and D.

To assess the extent of SARS-CoV-2 sequence intragenetic variability, we analyzed 15,224 publicly available high-throughput sequencing data sets from infected individuals (Table S1). The raw sequencing reads were mapped to the SARS-CoV-2 isolate Wuhan-Hu-1 reference genome, and the composition of each nucleotide at each position on the viral genome was generated. Consensus sequences were produced for each data set, and the nucleotide compositions for each position were compared to the respective consensuses. To reduce the number of variations due to amplification bias and sequencing errors, duplicated reads were combined, and only positions mapped with a sequencing depth of 50 reads and having at least 5 reads with variations compared to the sample consensus were considered. Overall, we identified 301,742 variations from 11,362 samples located on 26,113 positions of the 29,903-nucleotide (nt) SARS-CoV-2 genome. We observed an average of 26.6 ± 132.0 variable nucleotides per sample (ranging from 1 to 5,295 variations/sample) (Fig. 1A).

### Analysis of the type of intragenetic variations present in SARS-CoV-2 samples from infected individuals.

The analysis of the type of nucleotide changes within samples revealed that 52.2% were transitions (either purine→purine or pyrimidine→pyrimidine) and 47.8% were transversions (purine→pyrimidine or pyrimidine→purine). Notably, the highest nucleotide variations corresponded to C→U transitions (43.5%), followed by G→U transversion (28.1%) (Fig. 1B), both types encompassing 71.6% of all variations. Since editing by host enzymes depends on the sequence context, we extracted 2 nt upstream and downstream from each genomic position corresponding to variations and generated sequence logos. Our results indicated a high number of A’s and U’s around all variation types and sites (62.1% ± 3.4%) (Fig. 1B). However, no significant enrichment of base composition within the motifs surrounding the variations compared to the composition of the viral genome was observed (all Bonferroni-corrected *P* values were greater than 0.74, as determined using Fisher's exact test). Because SARS-CoV-2 is composed of 62% A/U, this suggests that the observed numbers of A’s and U’s around variation sites are mainly due to the A/U content of the viral genome and that no discernible motifs appear to be enriched around these sites. We are therefore unable to confirm whether these intragenetic variations are caused by host RNA editing enzymes.

### Identification of recurrent genetic variants of SARS-CoV-2 in samples from infected individuals.

**FIG 2:**
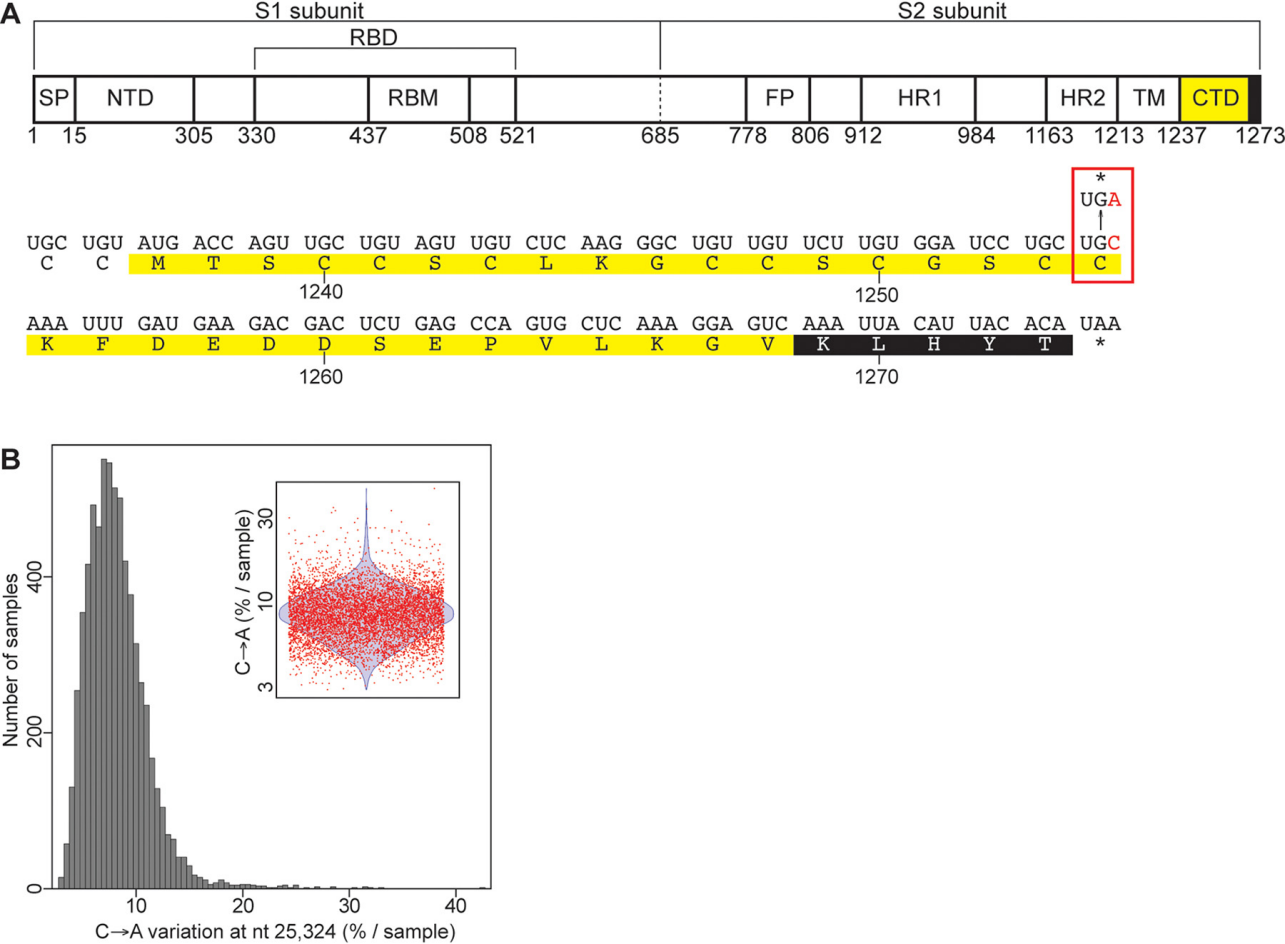
Localization of the C→A missense mutation on the SARS-CoV-2 S protein. (A) Schematic representation of the functional domain of the SARS-CoV-2 S protein. Below is shown the localization of the C→A variation on the carboxy-terminal domain (CTD) of the S protein. The mutation is colored and boxed in red. The carboxy-terminal domain (CTD) and the ERRS are colored in yellow and black, respectfully. (B) Distribution of the intrasample proportion of the C→A transversion at position 25,324 in the 6,668 samples containing this subspecies. The inset represents the distribution, using red dots to represent the samples having this intragenetic variation and a blue violon to show the distribution of the data.

To identify biologically relevant intragenetic variations, we examined the variable positions that are recurrent in the samples analyzed. The variable positions were tabulated for each sample, and then recurrent intragenetic variations were calculated as percentages of samples containing a variation at each position. Most variations are distributed homogeneously on the viral genome. The number of variations strongly correlates with the length of each gene (Pearson correlation coefficient of 0.972), and most are poorly shared among samples (Fig. 1C and D). However, our analysis reveals 15 recurrent intragenetic variations shared by at least 5% of the samples analyzed (Fig. 1C, above the blue line; Table 1). Among these, four transversions (at nt 25324, 25334, 25336, and 25337) located at the 3′ end of the S gene are the most recurrent variations (Fig. 1C, inset; Table 1). Three of these transversions (at nt 25334, 25336, and 25337) correspond to missense mutations: E1258D (46.4% of the samples), E1258Q (27.6% of the samples), and D1259H (20.1% of the samples). Interestingly, the most observed variation (at nt 25324) is shared by 58.7% of the samples (6,668 of the 11,362 samples) and corresponds to a C→A transversion producing a nonsense mutation at amino acid 1254 of the S protein (Fig. 1C and D, red lines; Fig. 2A, red rectangle). The resulting S protein lacks the last 20 amino acids (SΔ20), which includes the ERRS motif at its carboxy terminus (Fig. 2A, white letters on a black background). Among the samples with this intragenetic variation, this C→A transversion represents from 2.9 to 42.4% of the subspecies identified (mean of 8.2% ± 2.9%) (Fig. 2B; Table 1).

**TABLE 1:** Recurrent SARS-CoV-2 genome intragenetic variations shared by at least 5% infected individuals^*a*^

Position (nt)	Proportion of samples (%)	Type of variation	Gene	Amino acid	Codon		Amino acid		Context (−2 to +2)	Frequency distribution (% of population)			
					Consensus	Variant	Consensus	Variant		Mean	SD	Min	Max
25324	58.69	C→A	S	1254	UG**C**	UG**A**	Cys (C)	Stop	UG**C**AA	8.19	2.89	2.86	42.37
25336	46.37	A→C	S	1258	GA**A**	GA**C**	Glu (E)	Asp (D)	GA**A**GA	6.38	2.10	2.42	29.09
25334	27.57	G→C	S	1258	**G**AA	**C**AA	Glu (E)	Gln (Q)	AU**G**AA	4.76	1.63	2.03	22.81
25337	20.11	G→C	S	1259	**G**AC	**C**AC	Asp (D)	His (H)	AA**G**AC	4.68	2.12	2.07	28.57
29187	10.95	C→U	N	305	G**C**A	G**U**A	Ala (A)	Val (V)	UG**C**AC	3.35	2.53	1.81	46.91
29188	10.68	A→G	N	305	GC**A**	GC**G**	Ala (A)	Ala (A)	GC**A**CA	3.32	2.56	1.79	46.91
18591	10.21	C→G	ORF1ab	6108	GU**C**	GU**G**	Val (V)	Val (V)	GU**C**UU	3.78	0.85	2.54	7.96
11874	10.02	U→C	ORF1ab	3870	G**U**A	G**C**A	Val (V)	Ala (A)	AG**U**AG	4.21	2.27	2.08	38.55
15965	8.12	G→U	ORF1ab	5233	U**G**U	U**U**U	Cys (C)	Phe (F)	CU**G**UU	3.01	2.48	1.88	44.19
29039	7.95	A→U	N	256	A**A**G	A**U**G	Lys (K)	Met (M)	CU**A**AG	4.26	1.66	2.06	21.74
6696	7.19	C→U	ORF1ab	2144	C**C**U	C**U**U	Pro (P)	Leu (L)	GC**C**UU	3.59	3.31	1.92	48.85
28253	6.51	C→U	ORF8	120	UU**C**	UU**U**	Phe (F)	Phe (F)	UU**C**AU	8.58	7.98	1.86	48.42
635	5.18	C→U	ORF1ab	124	**C**GU	**U**GU	Arg (R)	Cys (C)	UU**C**GU	8.72	6.50	1.92	48.00
9502	5.17	C→U	ORF1ab	3079	GC**C**	GC**U**	Ala (A)	Ala (A)	GC**C**UU	3.98	3.29	1.99	49.40
25323	5.14	G→C	S	1254	U**G**C	U**C**C	Cys (C)	Ser (S)	CU**G**CA	4.27	1.84	2.12	16.95

a Frequency distributions were calculated to generate statistics on the intragenetic variant populations. The variations are sorted by their recurrence, with the most shared variation at the top of the table. The mutations are indicated by underlined boldface residues.

### Analysis of intragenetic variations present in SARS-CoV-2 samples from infected cells.

**FIG 3:**
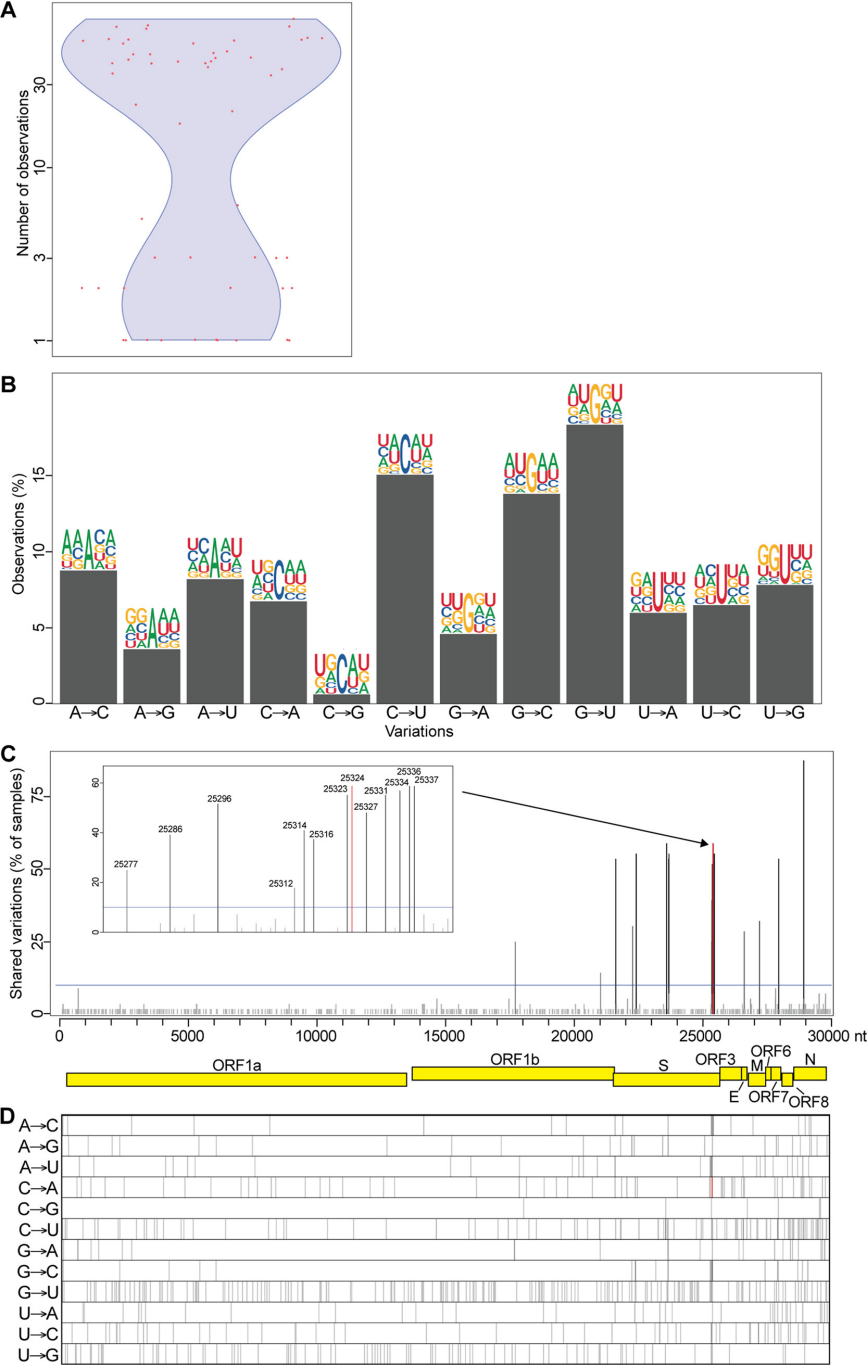
Intrasample variability of the SARS-CoV-2 genome in infected cells. (A) Number of intragenetic variations observed for each sample analyzed. The red dots represent the 65 samples analyzed, and the blue violon shows the distribution of the data. (B) Type of variation and sequence context for each intrasample variable position. Bars represent the percentage of each type. Sequence context is represented by logos comprised of the consensus nucleotides (center) with 2 nt upstream and 2 downstream from each intrasample variable position. (C) Recurrent intragenetic variations are represented as percentages of samples containing a variation at each position. The SARS-CoV-2 genome and its genes are represented by yellow boxes below the graph. The blue line indicates 10% shared variations and was used to extract the intrasample variations listed in Table 2. The inset represents a magnification of the cluster identified at the end of the S gene. (D) One-dimensional representation of the data shown in panel C for each type of variation individually. The location of the C→A variation at position 25,324 is indicated by a red line in panels C and D.

To further investigate variations in a more controlled system, we used 65 high-throughput sequencing data sets generated in a recent transcription profiling study of several cell lines infected with SARS-CoV-2 (29). As described above, the raw sequencing reads from infected cells were mapped to the SARS-CoV-2 genome sequence, the composition of each nucleotide at each position on the viral genome was generated, and nucleotide variations compared to respective consensus sequences were calculated (Fig. 3A). Because the sequencing depths of the samples were low, we considered positions mapped by at least 20 reads and having at least 2 reads with variations compared to the sample consensus. In the samples derived from infected cells, we observed 29.7% and 70.3% of transitions and transversions, respectively. Similar to observations in samples from infected individuals, the highest nucleotide variations corresponded to G→U transversions (26.1%) and C→U transitions (21.6%) (Fig. 3B). We then analyzed nucleotide compositions 2 nt upstream and downstream of the intragenetic variations. As described above, a high number of A’s/U’s (57.8% ± 7.7%) were present around variation sites (Fig. 3B), consistent with the 62% A/U composition of the SARS-CoV-2 genome, indicating no enrichment of sequence motifs around these sites, except for the expected high number of A’s and U’s.

We then examined the intragenetic variable positions that are recurrent among the cell lines analyzed. We identified 29 positions within the viral populations showing intragenetic variation enrichment in at least 10% of the cell cultures, and most of them are located on structural genes, which are carried on the last 3′-terminal third of the viral genome (Fig. 3C and D). Similar to our observation from the samples from infected individuals, a cluster of recurrent variations is located at the 3′end of the S gene, including the C→A transversion at position 25324 shared in 58.9% of the cell lines analyzed (Fig. 3C and D, red lines; Table 2). Overall, our results indicate consistent results between intragenetic variations observed in infected cell lines and in samples from infected individuals, including the presence of the viral subspecies resulting in an S protein truncated of its last 20 amino acids (SΔ20).

**TABLE 2:** Recurrent SARS-CoV-2 genome intragenetic variations shared by at least 10% of infected cell cultures^*a*^

Position (nt)	Proportion of samples (%)	Type of variation	Gene	Amino acid	Codon		Amino acid		Context (−2 to +2)	Frequency distribution (% of population)			
					Consensus	Variant	Consensus	Variant		Mean	SD	Min	Max
28853	82.14	U→A	N	194	**U**CA	**A**CA	Ser (S)	Thr (T)	GU**U**CA	8.16	7.99	1.52	35.42
25336	58.93	A→C	S	1258	GA**A**	GA**C**	Glu (E)	Asp (D)	GA**A**GA	21.77	5.81	12.00	42.22
25324	58.93	C→A	S	1254	UG**C**	UG**A**	Cys (C)	Stop	UG**C**AA	25.21	7.37	12.00	42.37
23525	58.93	C→U	S	655	**C**AU	**U**AU	His (H)	Tyr (Y)	AA**C**AU	8.36	3.35	3.49	16.67
25337	58.93	G→C	S	1259	**G**AC	**C**AC	Asp (D)	His (H)	AA**G**AC	20.43	4.65	12.86	35.56
25334	57.14	G→C	S	1258	**G**AA	**C**AA	Glu (E)	Gln (Q)	AU**G**AA	12.98	6.44	3.08	22.81
25381	55.36	A→C	S	1273	AC**A**	AC**C**	Thr (T)	Thr (T)	AC**A**UA	26.73	5.21	8.33	37.50
22343	55.36	G→C	S	261	**G**GU	**C**GU	Gly (G)	Arg (R)	CU**G**GU	6.51	2.84	2.27	13.79
25323	55.36	G→C	S	1254	U**G**C	U**C**C	Cys (C)	Ser (S)	CU**G**CA	9.03	4.13	2.82	17.24
25331	55.36	G→U	S	1257	**G**AU	**U**AU	Asp (D)	Tyr (Y)	UU**G**AU	6.35	2.94	2.60	13.33
27883	53.57	C→U	ORF7b	43	G**C**C	G**U**C	Ala (A)	Val (V)	CG**C**CU	6.74	2.43	2.40	11.19
27882	53.57	G→C	ORF7b	43	**G**CC	**C**CC	Ala (A)	Pro (P)	AC**G**CC	6.88	2.52	2.40	11.67
25296	51.79	A→C	S	1245	A**A**G	A**C**G	Lys (K)	Thr (T)	CA**A**GG	7.16	2.38	2.94	12.96
23606	51.79	C→U	S	682	**C**GG	**U**GG	Arg (R)	Trp (W)	CU**C**GG	31.65	12.73	3.95	48.15
25327	48.21	A→U	S	1255	AA**A**	AA**U**	Lys (K)	Asn (N)	AA**A**UU	5.31	2.23	2.60	9.43
23616	48.21	G→A	S	685	C**G**U	C**A**U	Arg (R)	His (H)	AC**G**UA	21.11	10.20	2.38	38.71
23616	44.64	G→C	S	685	C**G**U	C**C**U	Arg (R)	Pro (P)	AC**G**UA	21.11	10.20	2.38	38.71
21550	41.07	A→C	ORF1ab	7095	**A**AC	**C**AC	Asn (N)	His (H)	AC**A**AC	39.31	9.06	18.75	50.00
21551	41.07	A→U	ORF1ab	7095	A**A**C	A**U**C	Asn (N)	Ile (I)	CA**A**CU	38.79	9.19	18.75	50.00
25286	39.29	A→U	S	1242	**A**GU	**U**GU	Ser (S)	Cys (C)	GU**A**GU	4.05	1.27	2.63	7.84
25314	39.29	G→U	S	1251	G**G**A	G**U**A	Gly (G)	Val (V)	UG**G**AU	4.15	1.54	2.56	7.14
27134	32.14	U→C	M	204	UA**U**	UA**C**	Tyr (Y)	Tyr (Y)	UA**U**AA	3.17	1.16	1.87	5.75
22206	30.36	A→G	S	215	G**A**U	G**G**U	Asp (D)	Gly (G)	UG**A**UC	4.30	1.64	2.44	9.21
25316	30.36	U→C	S	1252	**U**CC	**C**CC	Ser (S)	Pro (P)	GA**U**CC	4.85	1.89	2.67	9.38
26542	28.57	C→U	M	7	A**C**U	A**U**U	Thr (T)	Ile (I)	UA**C**UA	11.69	15.28	1.96	47.01
25296	26.79	A→U	S	1245	A**A**G	A**U**G	Lys (K)	Met (M)	CA**A**GG	7.16	2.38	2.94	12.96
25277	25.00	A→U	S	1239	**A**GU	**U**GU	Ser (S)	Cys (C)	CC**A**GU	3.50	0.69	2.67	5.06
17641	25.00	G→A	ORF1ab	5792	**G**CU	**A**CU	Ala (A)	Thr (T)	CA**G**CU	4.28	1.83	2.56	9.09
25331	25.00	G→C	S	1257	**G**AU	**C**AU	Asp (D)	His (H)	UU**G**AU	6.35	2.94	2.60	13.33
25334	25.00	G→U	S	1258	**G**AA	**U**AA	Glu (E)	Stop	AU**G**AA	12.98	6.44	3.08	22.81
25323	23.21	G→U	S	1254	U**G**C	U**U**C	Cys (C)	Phe (F)	CU**G**CA	9.03	4.13	2.82	17.24
25316	19.64	U→G	S	1252	**U**CC	**G**CC	Ser (S)	Ala (A)	GA**U**CC	4.85	1.89	2.67	9.38
25312	17.86	U→G	S	1250	UG**U**	UG**G**	Cys (C)	Trp (W)	UG**U**GG	3.50	0.72	2.56	4.76
20956	14.29	C→U	ORF1ab	6897	**C**UU	**U**UU	Leu (L)	Phe (F)	AU**C**UU	14.13	14.84	2.38	35.48
21550	12.50	A→C	ORF1ab	7095	**A**AC	**C**AC	Asn (N)	His (H)	AC**A**AC	39.31	9.06	18.75	50.00
21551	12.50	A→U	ORF1ab	7095	A**A**C	A**U**C	Asn (N)	Ile (I)	CA**A**CU	38.79	9.19	18.75	50.00
25273	10.71	G→C	S	1237	AU**G**	AU**C**	Met (M)	Ile (I)	AU**G**AC	2.95	0.44	2.53	3.77

a Frequency distributions were calculated to generate statistics on the intragenetic variant populations. The variations are sorted by their recurrence, with the most shared variation at the top of the table. The mutations are indicated by underlined boldface residues.

### Increased fusogenic properties of SARS-CoV-2 SΔ20.

**FIG 4:**
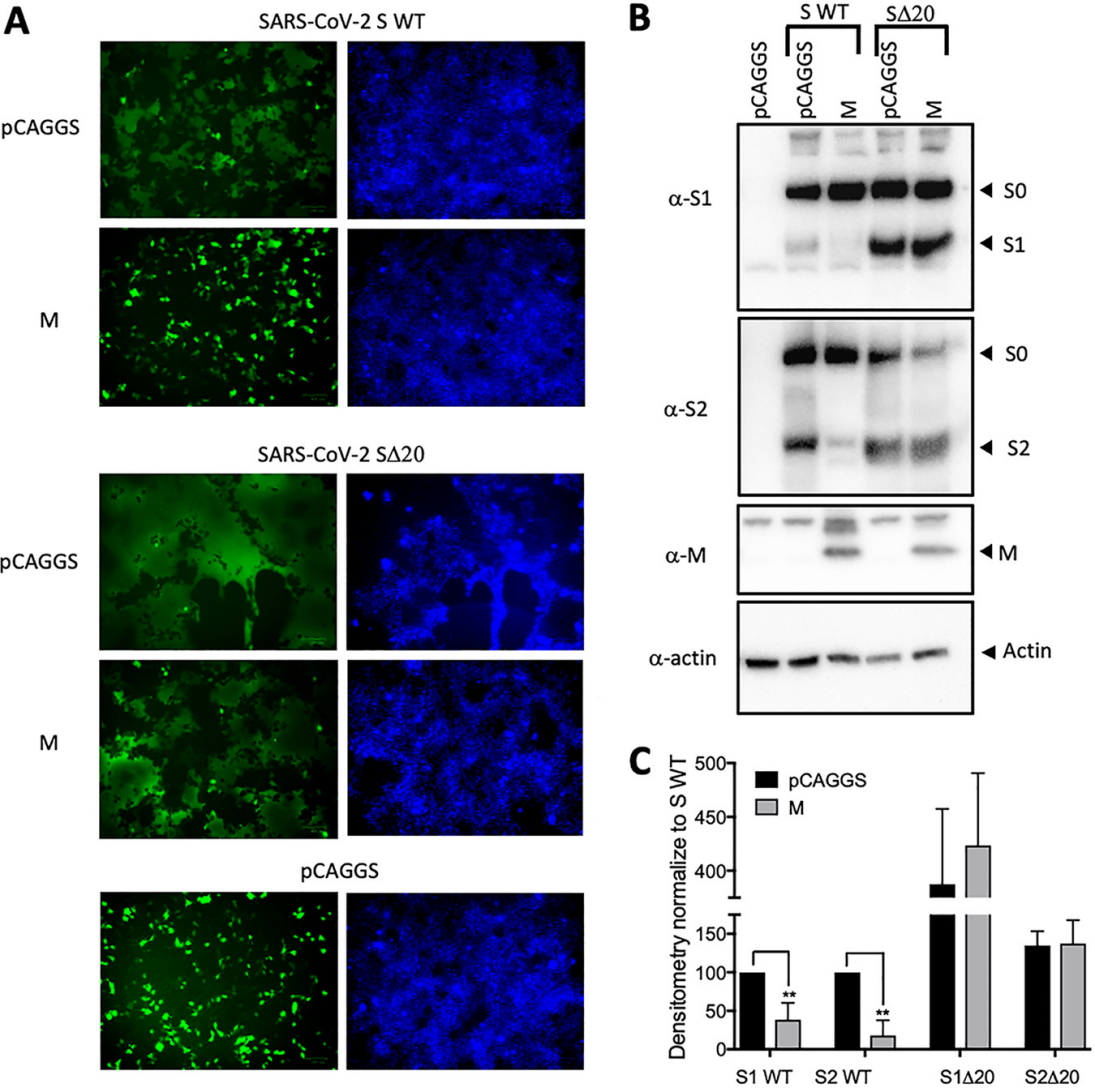
Increased processing and cytopathic syncytium formation by the SARS-CoV-2 SΔ20 protein. (A) Fluorescence microscopy of HEK-293T-hACE2 cells expressing GFP (green) with empty vector (pCAGGS) or plasmid expressing SARS-CoV-2 S or SARS-CoV-2 SΔ20 in the presence or absence of M protein. Counterstaining using Hoechst dye (blue), which labels nuclear DNA, is shown in the right panel. (B) Processing of spike protein was detected using anti-S1 and anti-S2 immunoblotting of HEK-293T cell lysates previously transfected with empty vector (pCAGGS) or vector expressing SARS-CoV-2 S or SARS-CoV-2 SΔ20 in the presence or absence of M protein. (C) Three independent immunoblots, as shown in panel B, were quantified using densitometry and statistically analyzed using a two-tailed Student's *t* test (**, *P* < 0.05).

**FIG 5:**
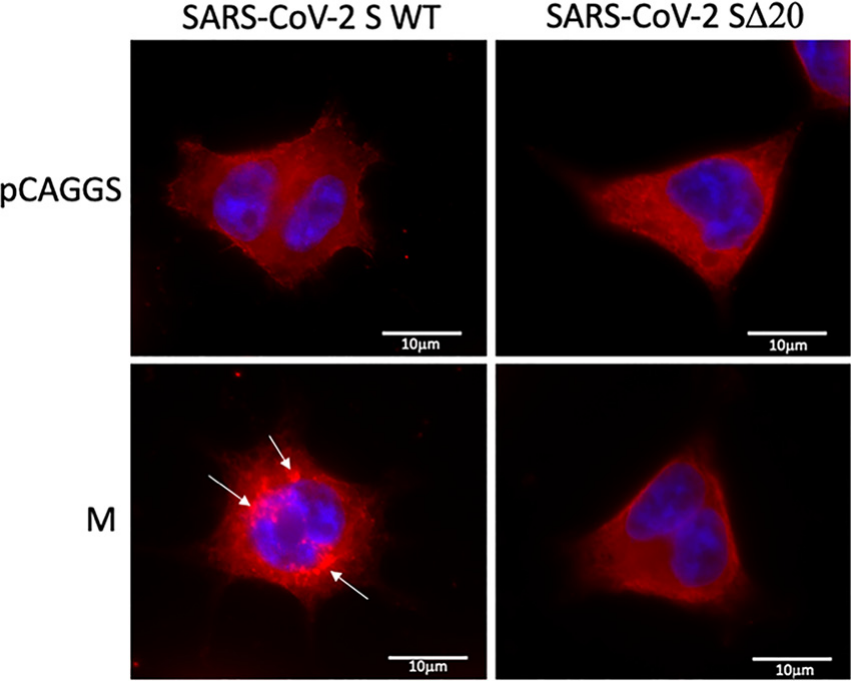
Subcellular localization of the S WT and SΔ20 in the presence or absence of M protein. HEK-293T cells expressing S proteins both with and without M protein were stained with anti-S protein (red), and the nucleus was stained with Hoechst 33342 dye (blue). Coexpression of M protein induced intracellular accumulation of the S WT (white arrow) but not the SΔ20 protein.

SARS-CoV-2 viral entry into cells is triggered by the interaction between the S glycoprotein and its cellular receptor, ACE2. While the complete mechanism of viral entry is not fully understood, it is known that S undergoes different processing steps by cellular surface and endosomal proteases. For several coronaviruses, the S protein mediates not only virion fusion but also syncytium formation (7, 12, 13). The presence of dysmorphic pneumocytes forming syncytial elements is a well-described feature of COVID-19 disease severity (30). One particularity of SARS-CoV-2 compared to SARS-CoV is the presence of an additional furin-like cleavage site at the S1/S2 interface. As a consequence, SARS-CoV-2-infected cells have a higher propensity to express activated S at the surface, which can fuse with other cells expressing the receptor ACE2 and form syncytia (30). The normal route of S trafficking involves an accumulation at the ERGIC, which is known to involve, at least in part, the interaction of the cytoplasmic portion of S with the M protein encoded by SARS-CoV-2. This interaction allows complex formation leading to virion formation at the ERGIC interface. The discovery of the SΔ20 variant missing a portion of the C terminus directed us to investigate the effect on cell fusion using a syncytium assay in the presence of the M protein. HEK-293T cells stably expressing the human ACE2 were cotransfected with plasmids encoding green fluorescent protein (GFP), the M protein and the wild-type (WT) or Δ20 S protein. Consistent with previous findings (7), we observed syncytium formation in the presence of the S WT and SΔ20, indicating induction of cell-to-cell fusion (Fig. 4A). We also observed larger syncytium formation with SΔ20 compared to S WT, which indicates increased fusogenic activity of this truncated variant. As expected, the coexpression of the M protein and S WT completely abolishes syncytium formation, which is a consequence of S being retained to the ERGIC. Strikingly, M protein failed to inhibit syncytium formation in the presence of SΔ20 (Fig. 4A). To evaluate the effect of the Δ20 truncation on spike protein processing, we coexpressed the M protein with WT or Δ20 S protein in HEK293T in the absence of ACE2 to avoid cell fusion. Cells were lysed 24 h posttransfection, and spike processing was assessed by probing for SARS-CoV2 S1 and S2 subunits by immunoblotting. As seen in Fig. 4B and quantified in Fig. 4C, the SΔ20 protein undergoes increased processing, as observed by the presence of more S1 and S2 subunits compared to S WT (Fig. 4B, lane 2 versus lane 4). The coexpression of the M protein reduces the processing of the S WT protein while not affecting SΔ20 processing, as observed by a reduction of the S1 fragment only for the S WT (Fig. 4B, lane 3 versus lane 5). Taken together, the results shown in Fig. 4 indicate that SΔ20 displays increased processing and syncytium formation compared to the wild-type S protein and the truncation removes an important regulatory domain involving the M protein. As discussed earlier, the S protein possesses an ER retrieval signal (ERRS) at its carboxy terminus, which is required for the S protein to interact with the M protein and accumulate at the ERGIC. Deletion of this sequence in SARS-CoV was shown to reduce ERGIC accumulation within the ERGIC. We observed the same phenotype with the SARS-CoV-2 SΔ20 (Fig. 5). When M protein was coexpressed, the majority of the S WT was retained intracellularly, with little detected on the cell surface. In contrast, the majority of SΔ20 was distributed throughout the cytoplasm and on the cell surface. This result is consistent with recent observations published by Boson et al. using the SΔ19 truncation mutant (31).

## DISCUSSION

Previous analyses of SARS-CoV-2 nucleotide variations indicated a high prevalence of C→U transitions, suggesting that the viral genome was actively evolving, and host editing enzymes, such as APOBECs and ADARs, might be involved in this process (23, 24). Although instructive on the role of host involvement in SARS-CoV-2 genome evolution, these studies were performed on consensus sequences (i.e., one per sample) and explore only part of the genetic landscape of this RNA virus. Here, we used a large number of high-throughput sequencing data sets to profile the intrasample sequence diversity of SARS-CoV-2 variants in both infected individuals and infected cell lines. We observed extensive genetic variability of the viral genome, including a high number of transversions, and identified several positions with recurrent intragenetic variability in the samples analyzed. Notably, most of the samples possessed a C→A missense mutation, producing an S protein that lacks the last 20 amino acids (SΔ20) and results in increased cell-to-cell fusion and syncytium formation.

Most intrasample variations are distributed homogeneously across the viral genome and are not conserved or recurrent among samples, and a large number of them are C→U or G→U mutations. Previous analyses of SARS-CoV-2 sequence variations proposed that host editing enzymes might be involved in coronavirus transition editing, based on results showing that C→U transitions occur within a sequence context reminiscent of APOBEC1-mediated deamination (i.e., [AU]C[AU]) (21–24). Here, we investigated nucleotide compositions at each variation site and observed a high number of A’s and U’s around all variation types and sites. However, since the SARS-CoV-2 genome is 62% A/U-rich, and similar percentages of A’s and U’s were observed around all variations, we concluded that no motifs are enriched around these variations in the viral subspecies analyzed here. Consequently, our results do not allow us to conclude the frequency of intrasample genetic variations caused by host RNA editing enzymes. Previous reports used consensus sequence variation analyses to suggest the involvement of editing enzymes (21–24). If host RNA editing enzymes have a major role in coronavirus genome editing, such modified variants will likely be very abundant in the quasispecies population and thus be reflected on the consensus sequence (i.e., >50% positional frequency). In our study, the variations in each data set were compared to their respective consensus sequence. This means that if RNA editing did occur at high frequency on a defined positional hot spot, it would not have been captured by our analysis method of the quasispecies but directly reflected on the consensus sequence. We did not analyze variations in consensus sequences as this was done previously for SARS-CoV-2 (23, 24).

Although it is possible that host RNA editing enzymes are responsible for the occurrence of some variations, C→U transitions and G→U transversions are also generally associated with nucleotide deamination and oxidation, respectively (32–39). It is common practice to thermally inactivate SARS-CoV-2 samples before performing RNA extractions, reverse transcription-PCR (RT-PCR), and sequencing (40). However, heating samples can result in free radical formation, such as 8-hydroxy-20-deoxyguanine (8-Oxo-dG), which could cause high levels of C→A and G→U mutations and promote the hydrolytic deamination of C→U (32–35, 37, 39, 41, 42). It was previously reported that these types of mutations occur at low frequency, that they are mostly detected when sequencing is performed on only one DNA strand, and that they are highly variable across independent experiments (34, 36). Consequently, the transversions observed in our analysis could be due to heat-induced damage, RNA extraction, storage, shearing, and/or RT-PCR amplification errors. However, we identified several positions with intrasample variability recurrent in several independent samples from both infected individuals and infected cells. They were detected at moderate to high frequencies, ranging from 2.5 to 39.3% per sample (Tables 1 and 2), and most were derived from paired-end sequencing (90.7% of the samples) in which the two strands of a DNA duplex were considered. Thus, it is likely that these variations are genuine and represent hot spots for SARS-CoV-2 genome intrasample variability.

Among the variable positions identified in infected cells, most of them are located in the last 3′-terminal third of the viral genome. These cells were infected with a large number of viruses (i.e., a high multiplicity of infection [MOI]) for 24 h (29). The presence of several variations at positions in the region coding for the main structural proteins likely reflects that this is a region with increased transcriptional activity due to the requirement of producing their encoded mRNAs from subgenomic negative-sense RNAs (8).

Interestingly, a cluster of variations located at the 3′end of the S gene was observed for the two data sets analyzed. They correspond to four transversions located at the 3′end of the S gene and are shared by a large proportion of the samples. Three of these correspond to missense mutations changing the charged side chains of two amino acids (E1258D, E1258Q, and D1259H). Notably, most of the samples possess a variability at position 25324, producing a nonsense mutation at amino acid 1254 of the S protein. The resulting protein lacks the last 20 amino acids (SΔ20) and thus does not include the ERRS motif at its carboxy terminus. For SARS-CoV-1, the ERRS domain accumulates the S protein to the ERGIC and facilitates its incorporation into virions (11). While the mechanism is not completely understood, mutation of the ERRS motif on S resulted in a failure to interact with the M protein at the ERGIC and rather resulted in trafficking of S to the cell surface. Deletion of this motif might cause the S protein of SARS-CoV-2 to accumulate to the plasma membrane and increase the formation of large multinucleated cells known as syncytia. Consistent with these observations, our results indicate larger syncytium formation with SΔ20 compared to the complete S protein. Moreover, we observed that the M protein failed to prevent SΔ20-induced syncytium formation, as observed with the WT S protein, which correlates with the role of the M protein in interacting with the spike and retaining it in ERGIC. Similar mutants (SΔ18, SΔ19, and SΔ21) were recently reported to increase both infectivity and replication of vesicular stomatitis virus (VSV) and human immunodeficiency virus (HIV) pseudotyped with SARS-CoV-2 S protein in cultured cells (43–46). Because these viruses bud from the plasma membrane (47, 48), an increased localization at this site would explain the selection of these deletion mutants in pseudotyped virions. However, such variants would unlikely be transmitted horizontally in naturally occurring CoV, where the budding site is the ERGIC (9).

Our findings indicate the presence of consistent intrasample genetic variants of SARS-CoV-2, including a recurrent subpopulation of SΔ20 variants with elevated fusogenic properties. It is tempting to suggest a link between SARS-CoV-2 pathogenesis and the presence of SΔ20, since severe cases of the disease were recently linked to considerable lung damage and the occurrence of syncytia (30, 49). Also, as observed for several enveloped viruses, syncytium formation could allow cell-to-cell spreading without virion production, which could facilitate not only viral dissemination but also immune evasion (50). Clearly, more investigation is required to better define the extent of SARS-CoV-2 variability in infected hosts and to assess the role of these subspecies in the life cycle of this virus. More importantly, further studies on the presence of SΔ20 and its link with viral pathogenicity could lead to better diagnostic strategies and design treatments for COVID-19.

## MATERIALS AND METHODS

### Analysis of intragenetic variability within SARS-CoV-2 samples.

A total of 15,289 publicly available high-throughput sequencing data sets were downloaded from the NCBI Sequence Read Archive (up to 10 July 2020). They comprise 15,224 data sets from infected individuals and 65 data sets from infected cell lines. Table S1 in the supplemental material includes all of the accession numbers. All data sets were derived from Illumina sequencing technology. The data sets from infected cells were generated by Blanco-Melo et al. (29). Duplicated reads were combined to reduce amplification bias and mapped to the SARS-CoV-2 isolate Wuhan-Hu-1 reference genome (NC_045512v2) using hisat2 (v.2.1.0) (51). For each data set, the consensus sequences and the frequency of nucleotides at each position were extracted from files generated by bcftools (v.1.10.2) of the samtools package (v.1.1) with an in-house Perl script (52, 53). All further calculations were performed in R. To reduce the number of variations due to sequencing errors and/or protocol differences, only positions mapped with a sequencing depth of 50 reads and having at least 5 reads with variations compared to the sample consensus were considered. Sequence logos were generated with the ggseqlogo package (v.0.1) (54).

10.1128/mBio.00788-21.1 TABLE S1 List of the 15,289 publicly available high-throughput sequencing data sets downloaded from the NCBI Sequence Read Archive (SRA) and used in this study. Only SRA numbers are listed. The 65 data sets from infected cells also include “(cells)” as a label and are listed at the end. Download Table S1, TXT file, 0.2 MB. Copyright © 2021 Rocheleau et al. 2021 Rocheleau et al. https://creativecommons.org/licenses/by/4.0/ This content is distributed under the terms of the Creative Commons Attribution 4.0 International license.

### Cell culture and plasmids.

Human embryonic kidney 293T (HEK-293T) cells were obtained from the American Type Culture Collection (ATCC CRL-11268) and maintained in Dulbecco’s modified Eagle’s medium (DMEM) supplemented with 5% fetal bovine serum (Fisher Scientific), 5% bovine calf serum (Fisher Scientific), 100 U/ml penicillin, and 100 μg/ml streptomycin (Fisher Scientific). HEK-293T cells stably expressing human ACE2 (HEK-293T-hACE2 cell line; BEI Resources) were cultured and maintained in DMEM (Corning) supplemented with 10% fetal bovine serum (Sigma), 100 U/ml penicillin, and 100 μg/ml streptomycin. All cells were cultured at 37°C in a humidified atmosphere containing 5% CO_2_. pCAGGS expressing the SARS-CoV-2 S protein (Wuhan-Hu-1; WT) was provided by Florian Krammer (Mount Sinai). SARS-CoV-2 SΔ20 was generated using overlapping PCR to introduce a termination codon at residue 1254. The expression construct encoding SARS-CoV-2 M was generated by PCR amplification of the M gene from pLVX-EF1alpha-SARS-CoV-2-M-2×Strep-IRES-Puro (a kind gift of Nevan Krogan, UCSF) and addition of a stop codon to remove the Strep (streptavidin) tag prior to cloning into pCAGGS.

### Syncytium formation assay.

Twenty-four-well plates were seeded with HEK-293T-hACE2 cells in complete medium to obtain 90% confluence the following day. Cells were then transiently cotransfected using JetPRIME (Polyplus Transfection, France) with plasmids encoding GFP (murine leukemia virus [MLV]-GFP, a kind gift of James Cunningham, Brigham and Women’s Hospital), SARS-CoV-2 S or SARS-CoV-2 SΔ20, and M or pCAGGS at a 0.15:0.2:0.65 ratio. Eighteen hours posttransfection, cells were imaged (ZOE fluorescent cell imager; Bio-Rad) for syncytium formation using the green channel to visualize fusion of GFP-positive cells as performed previously (55).

### Western blot analysis.

HEK-293T cells were transfected with the empty vector (pCAGGS), with SARS-CoV-2 S or SARS-CoV-2 SΔ20 and M, or with pCAGGS using JetPRIME at a 1:1 ratio. The following day, cells were washed once with cold phosphate-buffered saline (PBS) and lysed in cold lysis buffer (1% Triton X-100, 0.1% IGEPAL CA-630, 150 mM NaCl, 50 mM Tris-HCl, pH 7.5) containing protease and phosphatase inhibitors (Cell Signaling). Proteins in cell lysates were resolved on 4 to 12% gradient SDS-polyacrylamide gels (NuPage; Invitrogen) and transferred to polyvinylidene difluoride (PVDF) membranes. Membranes were blocked for 1 h at room temperature with blocking buffer (5% skim milk powder dissolved in 25 mM Tris, pH 7.5, 150 mM NaCl, and 0.1% Tween 20 [TBST]). Processing of spike protein was detected by immunoblotting using an anti-S1 antibody (SARS-CoV/SARS-CoV-2 spike protein S1 polyclonal; Invitrogen) and anti-S2 antibody (SARS-CoV/SARS-CoV-2 spike protein S2 monoclonal; Invitrogen). Overexpression of M was also detected by immunoblotting and using an anti-M antibody (rabbit anti-SARS membrane protein; Novus Biologicals). Membranes were incubated overnight at 4°C with the appropriate primary antibody in the blocking buffer. Blots were then washed in TBST and incubated with horseradish peroxidase (HRP)-conjugated secondary antibody for 1 h at room temperature (anti-mouse HRP and anti-rabbit HRP; both from Cell Signaling). Membranes were washed, incubated in chemiluminescence substrate (SuperSignal West Femto Maximum Sensitivity substrate; Thermo Fisher Scientific), and imaged using the ChemiDoc XRS+ imaging system (Bio-Rad). In some instances, the same membrane was stripped and reprobed for actin (monoclonal anti-β-actin; Millipore Sigma). Densitometry was performed using ImageJ software (56) and data analysis with Prism 8 (GraphPad).

### Immunofluorescence.

HEK-293T cells were transiently cotransfected using JetPRIME (Polyplus Transfection, France) with plasmids encoding SARS-CoV-2 S or SARS-CoV-2 SΔ20 and M proteins. Twenty-four hours posttransfection, an 18-mm poly-l-lysine (PLL)-coated glass coverslip was seeded with cells in complete medium to obtain a 25% confluence the following day. Cells were then stained with an anti-S2 antibody (SARS-CoV/SARS-CoV-2 spike protein S2 monoclonal; Invitrogen) and sandwiched with a goat anti-mouse IgG conjugated with Alexa Fluor 594 (Thermo Fisher Scientific). Nuclei were counterstained with Hoechst 33342 stain solution. Cells were imaged on a Zeiss Axio Observer D1 fluorescence microscope, and the image was analyzed using ImageJ software (56).
